# svist4get: a simple visualization tool for genomic tracks from sequencing experiments

**DOI:** 10.1186/s12859-019-2706-8

**Published:** 2019-03-06

**Authors:** Artyom A. Egorov, Ekaterina A. Sakharova, Aleksandra S. Anisimova, Sergey E. Dmitriev, Vadim N. Gladyshev, Ivan V. Kulakovskiy

**Affiliations:** 1 Belozersky Institute of Physico-Chemical Biology, Lomonosov Moscow State University, Leninskiye gory 1, Moscow, 119234 Russia; 2 Department of Medical Physics, Faculty of Physics, Lomonosov Moscow State University, Leninskiye gory 1-2, Moscow, 119991 Russia; 3 Vavilov Institute of General Genetics, Russian Academy of Sciences, Gubkina 3, Moscow, 119991 Russia; 4 Faculty of Bioengineering and Bioinformatics, Lomonosov Moscow State University, Leninskiye gory 1-73, Moscow, 119234 Russia; 5 Engelhardt Institute of Molecular Biology, Russian Academy of Sciences, Vavilova 32, Moscow, 119991 Russia; 6 Division of Genetics, Department of Medicine, Brigham and Women’s Hospital and Harvard Medical School, Boston, MA 02115 USA; 7 Institute of Mathematical Problems of Biology RAS - the Branch of Keldysh Institute of Applied Mathematics of Russian Academy of Sciences, Vitkevicha 1, Pushchino, 142290 Moscow Region Russia

**Keywords:** Genomic tracks, Visualization, Next-generation sequencing, High-throughput sequencing, Ribo-Seq, RNA-Seq, Python, Genome browser

## Abstract

**Background:**

High-throughput sequencing often provides a foundation for experimental analyses in the life sciences. For many such methods, an intermediate layer of bioinformatics data analysis is the genomic signal track constructed by short read mapping to a particular genome assembly. There are many software tools to visualize genomic tracks in a web browser or with a stand-alone graphical user interface. However, there are only few command-line applications suitable for automated usage or production of publication-ready visualizations.

**Results:**

Here we present svist4get, a command-line tool for customizable generation of publication-quality figures based on data from genomic signal tracks. Similarly to generic genome browser software, svist4get visualizes signal tracks at a given genomic location and is able to aggregate data from several tracks on a single plot along with the transcriptome annotation. The resulting plots can be saved as the vector or high-resolution bitmap images. We demonstrate practical use cases of svist4get for Ribo-Seq and RNA-Seq data.

**Conclusions:**

svist4get is implemented in Python 3 and runs on Linux. The command-line interface of svist4get allows for easy integration into bioinformatics pipelines in a console environment. Extra customization is possible through configuration files and Python API. For convenience, svist4get is provided as pypi package. The source code is available at https://bitbucket.org/artegorov/svist4get/

**Electronic supplementary material:**

The online version of this article (10.1186/s12859-019-2706-8) contains supplementary material, which is available to authorized users.

## Background

Next-generation sequencing gave birth to multiple high-throughput methods of the life sciences, many of which are based on mapping short sequence reads to an existing genome assembly. Visualization of mapped read densities and computationally derived genome signal tracks is one of the most routine tasks in bioinformatics sequencing data analysis. One approach is the usage of dedicated genome browsers. The most popular universal tools such as UCSC Genome Browser [1] or Zenbu [2] are web-based and allow interactive exploration of existing genome annotation along with uploaded user data. However, in some cases, it is not convenient to upload the user data to a remote server, and the data can be visualized and explored with the help of stand-alone applications with graphical user interface such as Integrated Genome Viewer [3] and Integrated Genome Browser [4], or even directly in the console environment [5]. Finally, there are hybrid approaches, for example, BioUML bioinformatics platform [6] provides genome browsing functionality in both web-based and stand-alone versions.

Web-based genome browsers are great for exploratory data analysis of processed public data, but stand-alone tools are better suited for generation of custom paper-quality graphics, and for exploring data from ongoing or private experiments. In addition, it is often convenient to have a programmatic way to generate multiple images for several genomic loci. There are several existing tools aimed specifically to solve this task via scripting or command-line interface (Table 1). Among the most advanced tools, there are Gviz [7] and ggbio [8], the Bioconductor R packages dedicated to the production of paper-quality figures of genomic tracks and annotations. Users preferring command-line utilities can use fluff [9] and ngs.plot [10]. These tools provide some advanced functions for data analysis but allow only a minimalistic approach to the visualization of genomic track segments in particular genomic windows. Here we present svist4get, the software tool allowing detailed paper-quality visualization of signal tracks along transcriptome annotation at a particular genomic location.

**Table 1:** An overview of existing programmatic visualization tools for genomic signal tracks

	svist4get	fluff	ngs.plot	ggbio	Gviz	ASCIIGenome
Command-line interface	yes	yes	yes	no	no	yes
Programming language	Python	Python	R, Python	R	R	Java
API	yes	no	no	yes	yes	no
Output	pdf, png	png	png, tiff	pdf, png	pdf, png	console (text)
Vector graphics output	yes	no	no	yes	yes	no
Reference		[9]	[10]	[8]	[7]	[5]

All the listed tools can function in Linux environment and support bed or bedGraph format for genomic signal tracks and gtf or gff for genomic annotation. Most of the tools are not focused on visualization of genomic windows and include advanced functions for data analysis or exploration.

## Implementation

Svist4get is implemented in Python 3 and uses multiple pypi packages (argparse, biopython [11], configs, reportlab, pybedtools [12], wand, wheel). Pypi ‘wand’ package and ImageMagick are utilized for pdf-to-png conversion. Svist4get was developed and tested in Linux environment. The python svist4get package is available in pypi (python3 -m pip install svist4get), the source code and example data are provided in Additional file 1. Details of svist4get installation are given in Additional file 2.

As input data, svist4get supports bedGraph format for genomic signals and gtf format of the genome annotation. As the output data, svist4get can generate vector graphics in pdf and export raster graphics in png. ImageMagick is used to provide raster (png) output.

Given a particular genomic window and a set of genomic signal tracks, svist4get automatically performs moving-average smoothing of the signal tracks, if necessary, taking into account the image width and the visible length of the genomic window. However, svist4get is a pure visualization tool, thus the technical data conversion and pre-processing, such as read depth normalization, should be performed with external tools, such as deeptools [13], bedtools [14], or UCSC utilities [15].

To facilitate application of svist4get in standard scenarios and data exploration, the command line interface covers several practical use cases that arise in transcriptomic studies, without additional effort for user-side scripting. Furthermore, svist4get provides a Python API allowing additional customization and programmatic usage from within a Python program. The use cases and examples of svist4get results are described in the next section.

## Results and discussion

Svist4get capabilities are demonstrated in [16], where figures were produced with svist4get Python API. Here we show several practical use cases of the command-line interface by visualizing particular genomic windows related to genes and transcripts using existing genome annotation. The command line parameters to reproduce the presented images are provided in Additional file 2.

**Fig. 1:**
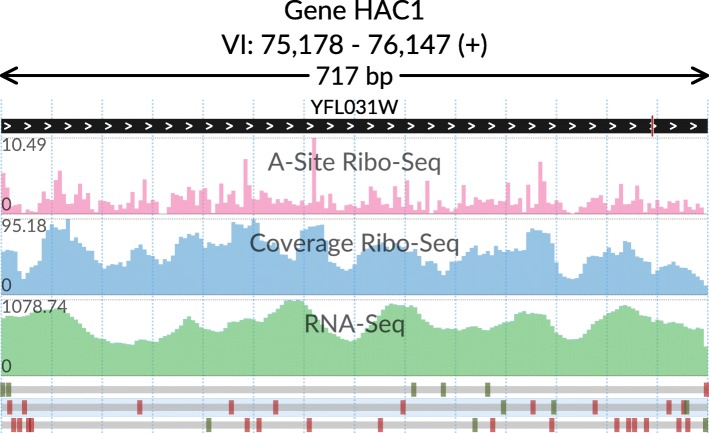
Transcript-centric selection and visualization of a genomic window. The top track shows the YFL031W transcript structure with the collapsed intronic region (short red bar on the right). The tracks in the middle show Ribo-Seq (ribosome A-sites and aggregated read density) and RNA-Seq (aggregated read density) signals. The bottom track shows the 0, + 1, and + 2 reading frames with the start and stop codons marked by green and red bars, respectively. The transcript open reading frame is highlighted. The data is taken from [17]

**Fig. 2:**
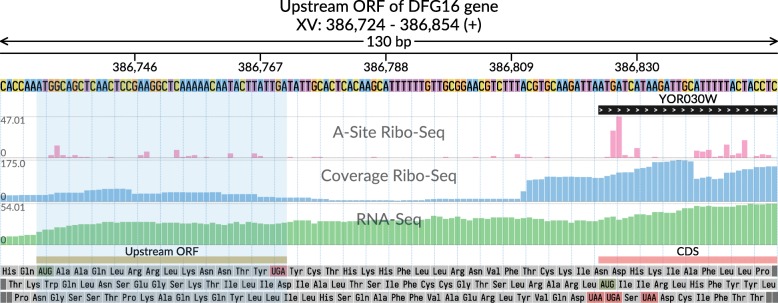
Ribo-Seq (ribosome A-sites and aggregated coverage) and RNA-Seq (aggregated coverage) signals in the vicinity of the translation initiation site of DFG16 gene. Upstream ORF in 5′ region is highlighted. In comparison to Fig. 1, the genomic window has lower length and the image uses a wider template, allowing single-nucleotide resolution. The data is taken from [17]

**Fig. 3:**
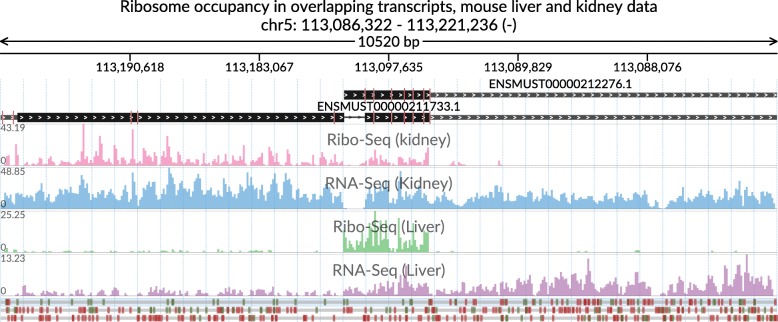
Ribo-Seq and RNA-Seq aggregated coverage signals in mouse kidney and liver data. The genomic window is centered on overlapping annotated transcripts displaying tissue-specific ribosome occupancy (Ribo-Seq tracks) and transcript abundance (RNA-Seq tracks). The red marks on the transcript structure track (on top) correspond to the collapsed intronic regions which are reconcilable for both shown transcripts. The data is taken from [20, 21]

The basic cases (Figs. 1 and 2) are illustrated by using yeast ribosome profiling (Ribo-Seq) and RNA-Seq data from [17] downloaded from GWIPs-viz [18], and yeast genome reference annotation from Ensembl [19]. For convenience, in the svist4get package, we include truncated sample data that is used for demonstration purposes. Visualization of tissue-specific expression of different transcript isoforms (Fig. 3) uses mouse Ribo-Seq data [20, 21] that was downloaded from GWIPs-viz [18, 22] and GENCODE M13 [23] mouse genome annotation.

### Basic visualization of genomic windows

We employed svist4get to generate a visualization of the genomic window containing the YFL031W transcript of HAC1 gene (Fig. 1). Based on genome annotation and a transcript identifier, svist4get selects a genomic window that includes a particular transcript. Alternative scenarios include the selection of a genomic window based on gene identifier and visualization of all transcripts in a given window (Additional file 2). Svist4get renders the transcript structure (based on genome annotation) as the top track, below it places the signal tracks (based on data in bedGraph format), and the structure of open reading frames (0, + 1, + 2, based on the nucleotide sequence of the displayed window) is shown at the bottom.

### Visualizing a genomic window at the single-nucleotide resolution

We also used svist4get to show a surrounding region of a translation initiation site of DFG16 yeast gene (Fig. 2), including an upstream open reading frame (ORF). The general layout of tracks in Fig. 2 is similar to that of Fig. 1. An additional track is used to show arbitrary genomic segments with user-defined labels (upstream ORF and CDS). A smaller genomic region surrounding DFG16 translation initiation site was selected based on transcript ID. A wider template (the predefined configuration file) allowed single-nucleotide resolution.

### Visualizing ribosome occ2upancy in overlapping transcripts

We also show a multi-track visualization illustrating differential ribosome occupancy in mouse kidney and liver Ribo-Seq data (Fig. 3). Reconcilable parts of introns of two annotated transcripts are collapsed (red vertical marks on the transcript structure tracks) to facilitate a non-interrupted view of the translated shortened open reading frame that is specific to the liver.

### Advanced features and customization

**Fig. 4:**
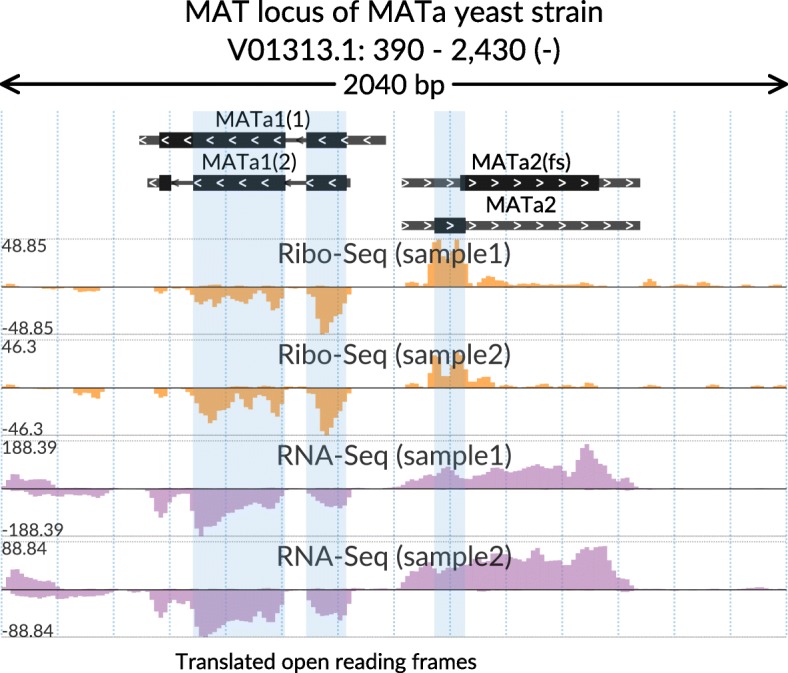
Ribo-Seq and RNA-Seq aggregated coverage of MAT locus in MATa yeast strain. Translated segments of transcripts are highlighted. Paired bedGraph tracks with custom colors are used to show coverage of two DNA strands separately. The data is taken from [16]

A basic bedGraph track is potentially useful to display various transcriptomic and genomic signals, such as DNase-Seq or ChIP-Seq. However, it is often necessary to visually separate signals on the primary and the reverse complementary DNA strands. To this end, svist4get provides paired bedGraph tracks, which use a single Y-axis to plot signals from a given pair of bedGraph files in the positive and negative value ranges (Fig. 4). Figure 4 also demonstrates multiple highlighting by showcasing translated segments of the MATa locus transcripts.

The visualization of svist4get is highly customizable. Some essential options, such as custom track coloring, are available directly through the command-line interface. Other parameters, such as color palette, bitmap DPI setting, font typeface, and page size are defined in configuration files (see Additional file 2 for details). The package includes default color palette and editable configuration files for generating figures to fit one- and two-column layout of an A4 page.

## Conclusions

Data from high-throughput sequencing requires specialized visualization tools. Here, we present svist4get, which produces publication-quality images of signal tracks along transcript structure in arbitrary genomic windows. We believe svist4get provides a reasonable compromise between tools with advanced R APIs and user-friendly graphical interfaces and can be useful as a component of bioinformatics pipelines as well as a stand-alone tool for data exploration.

## Availability and requirements

Project name: svist4get.

Project home page: https://bitbucket.org/artegorov/svist4get

Operating system(s): Linux.

Programming language: Python 3.

Other requirements: pypi packages (argparse, biopython, configs, reportlab, pybedtools, wand, wheel), ImageMagick (OS-level requirement for wand).

License: WTFPL http://www.wtfpl.net

## Additional files

Additional file 1: Svist4get source code and sample data. Python code and samples of yeast data used for generation of figures. (TGZ 5787 kb)

Additional file 2: Svist4get installation instructions and command-line examples. Installation instructions and console commands to reproduce the figures. (PDF 393 kb)
