# Supplementary material for: svist4get: a simple visualization tool for genomic tracks from sequencing experiments
Source: BMC Bioinformatics. 2019 Mar 6;20:113. doi: 10.1186/s12859-019-2706-8 (PMC6404320; doi:10.1186/s12859-019-2706-8)
Supplement: Supplementary file 2 — Svist4get installation instructions and command-line examples. Installation instructions and console commands to reproduce the figures. (PDF 393 kb) [file 12859_2019_2706_MOESM2_ESM.pdf]

SV4G

Svist4get installation instructions and command-line examples

Section 1. Installation 1

Post-installation step 2

Section 2. Console commands to reproduce the manuscript figures 2

Transcript-centric visualization (Figure 1 of the paper) 2

Visualization anchored at the translation initiation site (Figure 2 of the paper) 2

Section 3. Additional examples 3

Gene-centric selection of a genomic window 3

An arbitrary genomic window 3

Section 4. Advanced visualization of tissue-specific expression for overlapping transcripts in mouse (Figure 3 of the paper) 4

Section 5. Advanced features and customization (Figure 4 of the paper) 4

Useful customization options 5

Section 1. Installation

Svist4get is tested to work under Python 3.6 in Linux environment.

The stable release of svist4get can be installed directly from pypi:

```
python3 -m pip install svist4get
```

The development version is available at bitbucket:

```
git clone https://artegorov@bitbucket.org/artegorov/svist4get.git
cd svist4get
python3 -m pip install wheel
python3 setup.py sdist bdist_wheel
python3 -m pip install -e .
```

Note: the pdf files generated by svist4get can be imported and manually tweaked in vector graphics editing software such as Inkscape (inkscape.org), given the necessary fonts are installed in the user OS. Default fonts Iosevka (https://github.com/be5invis/Iosevka) and Lato (available at http://www.latofonts.com/lato-free-fonts/) are provided with svist4get.

Post-installation step

Svist4get comes with sample data that can be used to test visualization settings or reproduce example images.

The following command:

```
svist4get -sampledata
```

will unpack the sample data (`*.gtf, *.fa, *.bedGraph`) and editable example configuration files (`*.cfg` main configuration, `palette.txt` color palette) to the current working directory (see the newly created `svist4get_data` subfolder).

Section 2. Console commands to reproduce the manuscript figures

Figure 1 and Figure 2 of the svist4get paper can be easily reproduced by running particular commands in the console (assuming the package installation and the post-installation preparatory step are finished).

Transcript-centric visualization (Figure 1 of the paper)

Command line

```
svist4get -bg svist4get_data/RiboProElong_cut.bedGraph svist4get_data/RiboCov_cut.bedGraph
svist4get_data/mRNACov_cut.bedGraph -t YFL031W -gtf svist4get_data/S.cerevisiae.gtf -fa
svist4get_data/S.cerevisiae.dna.fa -it 'Gene HAC1' -bl 'A-Site Ribo-Seq' 'Coverage Ribo-Seq'
'RNA-Seq' -hi -hrf 1 -c A4_p1
```

Visualization anchored at the translation initiation site (Figure 2 of the paper)

Command line

```
svist4get -bg svist4get_data/RiboProElong_cut.bedGraph svist4get_data/RiboCov_cut.bedGraph
svist4get_data/mRNACov_cut.bedGraph -gtf svist4get_data/S.cerevisiae.gtf -fa
svist4get_data/S.cerevisiae.dna.fa -t YOR030W -w tis 100 30 -it 'Upstream ORF of DFG16 gene' -bl
'A-Site Ribo-Seq' 'Coverage Ribo-Seq' 'RNA-Seq' -gi 386730-386772 -gi 386824-386999 -gil
'Upstream ORF' 'CDS' -hf 386730 386772 ' ' -c A4_p2
```

Section 3. Additional examples

Gene-centric selection of a genomic window

The visualization shows intronic segment and another transcript in the selected window. An additional box highlight is used to specifically mark the intronic segment of the transcript.

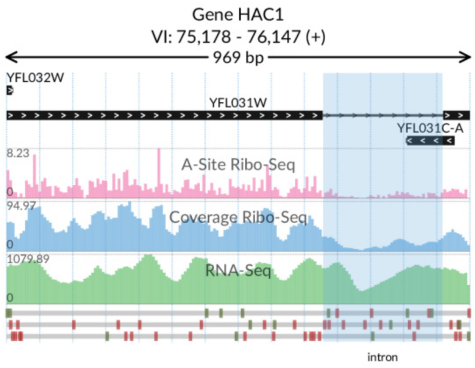

Command line

```
svist4get -bg svist4get_data/RiboProElong_cut.bedGraph svist4get_data/RiboCov_cut.bedGraph
svist4get_data/mRNACov_cut.bedGraph -g YFL031W -gtf svist4get_data/S.cerevisiae.gtf -fa
svist4get_data/S.cerevisiae.dna.fa -it 'Gene HAC1' -bl 'A-Site Ribo-Seq' 'Coverage Ribo-Seq'
'RNA-Seq' -hf 75839 76091 'intron' -c A4_p1
```

An arbitrary genomic window

Here we used svist4get to visualize an arbitrary genomic window on the chromosome I.

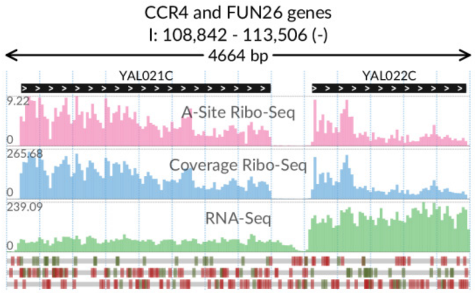

Command line

```
svist4get -bg svist4get_data/RiboProElong_cut.bedGraph svist4get_data/RiboCov_cut.bedGraph
svist4get_data/mRNACov_cut.bedGraph -gtf svist4get_data/S.cerevisiae.gtf –fa
svist4get_data/S.cerevisiae.dna.fa -bl 'A-Site Ribo-Seq' 'Coverage Ribo-Seq' 'RNA-Seq' -w I
108842 113506 -it 'CCR4 and FUN26 genes' -rc -c A4_p1
```

Section 4. Advanced visualization of tissue-specific expression for overlapping transcripts in mouse (Figure 3 of the paper)

The necessary data are not included in the svist4get samples and can be downloaded from [https://gwips.ucc.ie/downloads/index.html] (Ribo-Seq and RNA-Seq data of mouse liver and kidney, 'Liver: Janich15 ZT12' and 'Kidney: CasteloSzekely17 ZT12'), [ftp://ftp.ebi.ac.uk/pub/databases/gencode/Gencode_mouse/release_M19/gencode.vM19.annotation.gtf.gz] (the mouse genome annotation), [ftp://ftp.ebi.ac.uk/pub/databases/gencode/Gencode_mouse/release_M19/GRCm38.primary_assembly.genome.fa.gz] (the mouse genome assembly).
The bigWig (*.bw) files should be then converted to bedGraph format by custom scripts or UCSC utilities.

Possible command line

```
svist4get -bg Kidney_Footprints.bedGraph Kidney_RNA_Seq.bedGraph Liver_Footprints.bedGraph
Liver_RNA_Seq.bedGraph -gtf data_sv4g/Mouse.gtf -fa data_sv4g/Mouse.fa -g ENSMUSG00000051339.10
-st ENSMUST00000211733.1 ENSMUST00000212276.1 -hi –rc -it 'Ribosome occupancy in overlapping
transcripts, mouse liver and kidney data' -bl 'Ribo-Seq (Kidney)' 'RNA-Seq (Kidney)' 'Ribo-Seq
(Liver)' 'RNA-Seq (Liver)' -c A4_p2 -hrf 0
```

Section 5. Advanced features and customization (Figure 4 of the paper)

Figure 4 can be reproduced by running the following commands in the console (assuming the package installation and the post-installation preparatory step are finished).

Command line

```
svist4get -gtf svist4get_data/MATa.gtf -fa svist4get_data/MATa.fasta -pbg
svist4get_data/mata_ribo1_plus.bedGraph svist4get_data/mata_ribo1_minus.bedGraph -pbg
svist4get_data/mata_ribo2_plus.bedGraph svist4get_data/mata_ribo2_minus.bedGraph -pbg
svist4get_data/mata_rna1_plus.bedGraph svist4get_data/mata_rna1_minus.bedGraph -pbg
svist4get_data/mata_rna2_plus.bedGraph svist4get_data/mata_rna2_minus.bedGraph -w V01313.1 390
2430 -rc -it 'MAT locus of MATa yeast strain' -bl 'Ribo-Seq (sample1)' 'Ribo-Seq(sample2)' 'RNA-
Seq (sample1)' 'RNA-Seq (sample2)' -bgc brightorange brightorange purple purple -c
svist4get_data/alt_A4_p1.cfg -hf 1224 1305 ' ' -hf 1533 1638 'Translated open reading frames' -hf
1692 1932 ' '
```

Useful customization options

Figure 4 of the manuscript used several advanced features: paired bedGraph tracks (`-pbg`), multiple highlighting (several `-hf`), and custom tracks colors (`-bgc`). Additional colors are supported through alternative `palette.txt` file which is referenced in the alternative configuration file `alt_A4_p1.cfg`. Two default configuration files (`A4_p1.cfg` and `alt_A4_p2.cfg`) provide default presets for generating figures to fit one- and two-column layout of an A4 page. Custom page size can be also defined, if necessary, along other useful tweaks such as image title show/hide switch (`show_title`, default = 1) and DPI setting for png export (`png_dpi`, default = 300).

The default configuration files can be found in the svist4get sample data folder which is unpacked at the post-installation step (see Section 1).

Detailed description of the configuration file parameters can be found online at https://bitbucket.org/artegorov/svist4get/
